# Oral *Candida* colonization and anti-fungal susceptibility pattern in patients with hematological malignancy

**DOI:** 10.18502/cmm.8.3.11210

**Published:** 2022-09

**Authors:** Maryam Talebshoushtari Zadeh, Ensieh Lotfali, Mahsa Fattahi, Sara Abolgasemi

**Affiliations:** 1 Infectious Diseases and Tropical Medicine Research Center, Shahid Beheshti University of Medical Sciences, Tehran, Iran; 2 Department of Medical Parasitology and Mycology, School of Medicine, Shahid Beheshti University of Medical Sciences, Tehran, Iran; 3 Center for Research and Training in Skin Diseases and Leprosy, Tehran University of Medical Sciences, Tehran, Iran

**Keywords:** *Candida*, *C. albicans*, *C. glabrata*, Hematologic malignancy

## Abstract

**Background and Purpose:**

Candidiasis is regarded as one of the most important fungal infections and a cause of disease and mortality in patients with hematological malignancy. Accordingly, antifungal prophylaxis is of significant importance in this regard. This study aimed to identify the epidemiology of *Candida* colonization and evaluate its antifungal susceptibility pattern in patients with hematological malignancy.

**Materials and Methods:**

In this study, the samples were collected from the oral cavity of 100 patients, and *Candida* colonization was confirmed by fungal culture. *Candida* strains were also identified by ITS-PCR. *In vitro* antifungal susceptibility tests against fluconazole, amphotericin B, and caspofungin were performed according to CLSI M60.

**Results:**

Demographic characteristics, comorbidities, distribution of *Candida* species (spp.), and antifungal susceptibility were analyzed in this study. The study participants included 100 patients with a mean age of 15.48%±48.74 years (age range: 17-84 years). Regarding gender distribution, the majority (64%) of the patients were male. In terms of the distribution of underlying hematologic malignancy, 27% of the cases had lymphoma. The most commonly isolated species among patients were *C. albicans* complex (49%; n=49), *C. glabrata* (39%; n=39), and co-colonization of *C. albicans* complex and *C.* with *C. glabrata* (10%; n=10). The overall resistance of *C. albicans* complex was 5% to fluconazole (n=5) and 2% to amphotericin B (n=2). Furthermore, *C. glabrata* showed 11% (n=11) resistance to fluconazole and was susceptible to amphotericin B. All *Candida* spp. isolated from patients who were susceptible to caspofungin.

**Conclusion:**

The high rate of colonization of *Candida* spp., especially the significant increase in the frequency of *C. glabrata* in patients with blood malignancies and the gradual increase in resistance to fluconazole, necessitate a change in the use of antifungal drugs for the prevention and experimental treatment of hematological malignancy.

## Introduction

Invasive fungal infections (IFIs) or invasive fungal diseases (IFD) are the most important causes of death from 29% to 90% [ 1 - 3 ]. Oral candidiasis is a fungal infection in the mouth caused by *Candida* species (spp.). Chemotherapy is one of the most important predisposing factors for fungal infections in malignancies [ 4 ]. With prolonged colonization during chemotherapy, the likelihood of invasive candidiasis with the same colonized isolates increases [ 5 ]. 

Azole-resistant *Candida* colonization may affect mortality since IFIs with the same organism could occur [ 6 ]. Fungal infection is one of the most important causes of disease and mortality in patients with hematologic malignancies [ 1 , 3 , 7 , 8 ]. The risk of IFIs in these patients is increasing [ 8 ], and despite recent advances in the diagnosis and management of IFD, high morbidity and mortality rates are still reported [ 6 , 9 , 10 ]. Hematologic malignancies mainly include acute leukemia and lymphomas, as well as multiple myeloma. These malignancies with or without neutropenia are risk factors for IFD [ 5 , 6 ]. Although *C. albicans* is considered to be the leading cause of most *Candida*-related IFDs, a shift toward non-albicans infections has been identified. Among the various species of non-*albicans*, some are concerned, such as *C. glabrata* and *C. parapsilosis*, due to different levels of antifungal resistance [ 11 ]. The evaluation of different fungal species is essential in fungal infections. Identification and detailed analysis of species susceptible to antifungal drugs, especially in immunosuppression patients are considered to determine the appropriate treatment protocol. This study aimed to assess oropharyngeal *Candida* colonization and anti-fungal susceptibility pattern in patients with hematological malignancy.

## Materials and Methods

### Study design

This applied cross-sectional study was conducted on 100 patients admitted to the Hematology Ward of Taleghani Hospital, Tehran, Iran, in 2021. Risk factors including demographic characteristics and underlying diseases (history of diabetes mellitus, immunodeficiency diseases, and rheumatic diseases, as well as head and neck radiotherapy) were collected through interviews with patients or clinical evidence. The samples were collected using swaps from the dorsal surface of the tongue and oropharynx. Patients with a history of non-hematologic malignancies were excluded from the study. 

### Identification of species

Species were cultured on Chrom agar medium (Sigma-Aldrich; USA) at 35°C for two days. PCR was used to differentiate between *C. albicans* complex and non-*C. albicans* spp. DNA was extracted from fresh 24-hour-colony cultures according to the protocol. The final solution (DNA pattern) was kept at -20°C until use. For the identification of non-*C. albicans* strains, the ITS regions of the isolated rDNA gene were amplified using the following primers [ 12 ]:

ITS1 (5’-TCCGTAGGTGAACCTGCGG-3’) 

ITS4 (5’- CCTCCGCTTATTGATATGC-3’)

### Antifungal susceptibility testing

Antifungal susceptibility testing was performed according to the CLSI-M60 protocol and the method described previously [ 13 ].

Before susceptibility testing, all isolates were cultured on Sabouraud dextrose agar (SDA; Merck, Germany) media, and the tests were performed in 96-well microplates. The final concentration of yeast suspensions was 0.5×10^3^ to 2.5×10^3^ CFU/mL. All antifungal drugs were purchased from Sigma-Aldrich (USA). Serial two-fold dilutions of fluconazole (FCZ), amphotericin B (AMB), and caspofungin (CAS) were prepared with RPMI 1640 medium (Invitrogen, Gibco) and buffered to pH=7.0 with 0.165 M Morpholine Propane Sulfonic Acid (MOPS) buffer (Sigma, USA) [ 14 ]. Yeast suspensions were diluted into RPMI in microplates incubated at 35°C for 48 h. Subsequently, the minimum inhibitory concentration (MIC) was determined, and the MIC breakpoints of antifungal agents were defined as follows: CAS (susceptible MIC≤0.25 µg/mL; resistant MIC≥1 µg/mL), FCZ (susceptible MIC≤8 µg/mL; resistant MIC>8 µg/mL), and AMB (susceptible MIC≤1 µg/mL; resistant MIC>1 µg/mL)[ 14 ].

*Candida parapsilosis* (ATCC 22019) was used as quality control. It is worth mentioning that all tests were carried out in triplicate.

### Statistic analysis

Quantitative data were expressed as mean±SD, and the percentage was used for stratified qualitative variables. Comparisons between quantitative variables were performed by t-test or Mann-Whitney test, and the qualitative variables were compared utilizing the Chi-square test. The data were analyzed in SPSS software (version 23), and a P-value of ≤0.05 was considered statistically significant. 

### Ethical approval

The project received approval from the Ethics Committee of Shahid Beheshti University of Medical Sciences, Tehran, Iran (code: IR.SBMU.MSP.REC. 1400.070).

## Results

### Demographic Data

This study was conducted on 100 patients with a mean age of 15.48%±48.74 years (age range: 17-84 years). Regarding gender distribution, the majority (64%) of the patients were male. In terms of the distribution of underlying hematologic malignancy, 27% of the cases had lymphoma. Furthermore, 20% of the cases were involved in neutropenia, and 4% of the participants had underlying diabetes mellitus

### Distribution of Candida species

*Candida* spp. were distributed as *C. albicans* complex (n=49), *C. glabrata* (n=31), co-colonization of *C. albicans* complex and *C. glabrata* (n=10), simultaneous colonization with *C. glabrata* and *C. parapsilosis* (n=4), colonization with *C. glabrata* and *C. tropicalis* (n=4), and colonization with *C. tropicalis* (n=2). The types of hematologic malignancies in the studied patients are presented in Table 1, and the highest frequency is related to lymphoma (n=27). There is a significant relationship between hematologic malignancy and colonized fungal species (*P=0.03*).

**Table 1:** Distribution of patients based on hematological malignancy and fungal species.

Hematological Malignancies	Candida species		
	*C. tropicalis* (%)	*C. albicans complex* (%)	*C. glabrata* (%)
ALL	0	7	4
AML	0	10	11
CLL	1	0	6
CML	0	3	3
ITP	0	1	0
MDS	0	1	2
MM	0	9	3
PNH	0	1	0
Aplastic Anemia	0	1	1
Hemolytic Anemia	0	2	0
Lymphoma	1	10	16
Myelofibrosis	0	1	1
Workup for Malignancy	0	3	3
Total	2	49	49

### Antifungal prophylaxis

The most commonly used drug for prophylaxis was FCZ (26%) and 53% of patients did not receive any prophylaxis. There is a significant relationship between the use of FCZ as prophylaxis and colonization of each species (*P=0.000*) and *C.glabrata* was the most common specieces among whom receiving FCZ prophylaxis. In total, 47% of patients received antifungal prophylaxis, which included FCZ (26%) and voriconazole (VCZ) (11%). The resistance and susceptibility of each cloned species to FCZ, CAS, and AMB are presented in Table 2.

**Table 2:** Anti-fungal susceptibility pattern in patients with hematological malignancy

*Candida* species	Antifungal	Breakpoints, µg/mL	Number of Isolates
*C. albicans* complex (n = 49)	Fluconazole	R≥8	5
		SDD=4	11
		S≤2	33
	Amphotericin B	R>1	2
		I	10
		S≤1	37
	Caspofungin	R≥1	0
		I=0.5	0
		S≤0.25	49
*C. glabrata* (n = 49)	Fluconazole	R≥64	11
		SDD≤32	38
		NM	0
	Amphotericin B	R>1	0
		I	3
		S≤1	46
	Caspofungin	R≥0.5	0
		I=0.25	0
		S≤0.12	49
*C. tropicalis* (n = 2)	Fluconazole	R≥8	0
		SDD=4	0
		S≤2	2
	Amphotericin B	R>1	0
		I	1
		S≤1	1
	Caspofungin	R≥1	0
		I=0.5	0
		S≤0.25	2

*C. albicans* complex (5%) and *C. glabrata* (11%) resistance to FCZ is significant. The most common infections by various fungal species were due to *C. albicans* complex (49%), *C. glabrata* (31%), simultaneous *C. albicans* complex and *C. glabrata* (10%), simultaneous *C. glabrata* and *C. parapsilosis* (4%), *C. glabrata* and *C. tropicalis* (4%), and *C. tropicalis* (2%). Among all patients, 15% had a history of bone marrow transplantation in which *C. glabrata* complex (6%) and *C. albicans* (9%) were isolated. In patients without a history of transplantation, 43% *C. glabrata*, 40% *C. albicans*, and 2% *C. tropicalis* were detected. There was a significant relationship between the presence and absence of a history of transplantation and the colonized species.

## Discussion

Invasive *Candida* infections are the most common types of IFD in hematologic cancer centers without the supervision of antifungal prophylaxis [ 15 , 16 ]. Fungal infections are life-threatening and common in patients with hematologic malignancies. Despite the use of various antifungal drugs in such patients, due to their immunosuppressive status, especially in cases undergoing chemotherapy or transplantation, as well as late diagnosis of such infections, we face significant morbidity and mortality among these patients seeking fungal infections. This is especially common at older ages. Identification of fungal infections in patients with hematologic malignancies, as well as antifungal drugs with the highest drug sensitivity, is essential to preserve the lives of many of these patients. In this regard, this study evaluated *Candida* colonization in patients with various hematologic malignancies admitted to the Hematology Ward of Taleghani Hospital, Tehran, Iran, in 2021. In our study, 20% of the patients were involved with neutropenia which is a serious complication that can occur during the treatment of hematologic malignancies and sometimes be due to fungal infection [ 17 ]. 

For high-risk patients, long-term neutropenia (decreased neutrophil count for at least 10 consecutive days) is the most important risk factor for the development and spread of fungal infections [ 9 ]. The probability of developing fungal infections after the diagnosis of acute leukemia is estimated to be about 11% in 100 days [ 11 ]. Patients with hematologic malignancies have been diagnosed with 35% mortality due to fungal infections [ 10 ]. In a study in Australia, 1.33% of patients with chronic lymphocytic leukemia developed IFD within 12 months of being diagnosed with cancer [ 18 ]. The literature concerning the epidemiology of fungal infection in patients with hematologic malignancies in the Asia-Pacific region is limited [ 3 , 7 , 8 ]. The increase in the incidence of fungal infections in these patients has been associated with increases in hospitalization time and the cost of treatment[ 19 ]. Continuous monitoring of changes in the epidemiology of IFDs as an important part of antifungal monitoring programs requires regular and accurate evaluation. This assessment is especially important in high-risk environments, such as oncology centers [ 11 ]. In this study, the most common fungal species detected were *C. albicans* complex, followed by *C. glabrata* and *C. glabrata* colonization increased by prophylactic interventions. Hamzavi et al. evaluated *Candida* colonization using the samples of oral, urine, nasal, and stool in pediatric patients with hematologic malignancies. In this study, the frequency of *C. albicans* complex was higher than that of other species (60%) [ 11 ].

In a study performed by Mardani et al., the frequency of *C. albicans* was estimated at 91.6% [ 4 ], and the frequency of *Candida* spp. was higher than that of other species in a study performed by Keymaram et al. [ 20 ]. Oropharyngeal *Candida* colonization was more prevalent in patients with acute myeloid leukemia, compared to healthy adults, and the dominant species was *C. glabrata* in a study carried out by Shirazian et al. conducted from 2016 to 2017 in Tehran, Iran [ 21 ].

Prophylactic antifungal treatment is effective in a wide range of organisms and is available intravenously and orally [ 22 ]. These species show higher levels of resistance to antifungal interventions and are associated with higher mortality from *C. albicans* [ 23 , 24 ]. In our study, 46% of patients received antifungal prophylaxis, and FCZ obtained the highest consumption among other prophylaxis (26%). The proper use of antifungals in vulnerable populations is important for the treatment of IFI [ 25 , 26 ]. 

The findings of a study by Sakamoto et al. show that the optimal prophylactic dose of FCZ in patients with hematologic malignancy is needed for the complete prevention of invasive candidiasis [ 27 ]. The prognostic model identifies patients at high risk for FCZ failure, indicating the importance of paying more attention to personalizing candidiasis treatment [ 28 ].

Epidemiological trends indicate the prevalence of non-*albicans Candida* in patients with hematologic malignancy [ 29 ]. In the last decade, an increase was observed in the prevalence of *C. glabrata* fungi in some cases [ 26 ]. In this study, *C. glabrata's* resistance to prophylactic interventions was significant, compared to a previous study in the same center [ 5 ] where *C. albicans'* resistance to FCZ was 2.7%. In the present study, this ratio was obtained at 5%. In a previous study, there was no *C. glabrata* resistance to FCZ; however, in the present study, increased drug resistance to FCZ was observed (11%). The main reason for these changes could be the use of FCZ as the first-line treatment in these patients, the emergence of resistance due to the use of broad-spectrum antibiotics, and the utilization of potent malignancy treatment protocols. Tracing serious infections and developing guidelines for infection control strategies and antifungal monitoring programs are essential [ 23 ]. Antifungal drugs, such as FCZ, itraconazole, posaconazole, CAS, and micafungin are all recommended with many consensus and guidelines for antifungal prophylaxis. Accordingly, it is of crucial significance to predict IFD classification and review new drugs, pharmacokinetics, as well as integrated, more scientific, appropriate, and effective prevention routes [ 30 ]. One of the limitations of our study was the small number of patients, which reduced the power of statistical comparisons.

## Conclusion

The high frequency of colonization of *Candida* spp., especially the significant increase in the frequency of *C. glabrata* in patients with hematologic malignancies and the gradual increase in FCZ resistance, changes in the use of antifungal drugs for prophylaxis, and empiric therapy in hematologic malignancies should be considered. Therefore, risk factors for species distribution, patterns of antifungal susceptibility of *Candida* isolates that cause candidiasis, and mortality risk factors should be evaluated and considered for more effective outcomes in antifungal therapy.

## Acknowledgments

None.

## Authors’ contribution

S.A. and M. TS. designed the research, collected the data, interpreted data, drafted, and revised the manuscript. E.L. and M.F. performed the laboratory tests and reviewed the analyses and the final version of the manuscript. S.A. interpreted the data, revised the manuscript for important intellectual content, and approved the final version. All authors have read and approved the manuscript. 

## Conflicts of interest

Not Applicable.

## Financial disclosure

This study was financially supported by the Infectious Diseases and Tropical Medicine Research Center, Shahid Beheshti University of Medical Sciences (SBMU), Tehran, Iran.
